# Selective nanosecond laser removal of retinal pigment epithelium for cell therapy

**DOI:** 10.1038/s41598-024-69917-z

**Published:** 2024-08-21

**Authors:** Van Phuc Nguyen, Athanasios J. Karoukis, Justin Hu, Zhuying Wei, Dongshan Yang, Abigail T. Fahim, Xueding Wang, Yannis M. Paulus

**Affiliations:** 1 Department of Ophthalmology, Johns Hopkins University School of Medicine, 600 North Wolfe Street, Baltimore, MD 21287 USA; 2 Department of Ophthalmology and Visual Sciences, University of Michigan, Ann Arbor, MI 48105 USA; 3 Department of Biomedical Engineering, University of Michigan, Ann Arbor, MI 48105 USA; 4 Center for Advanced Models for Translational Sciences and Therapeutics, University of Michigan, Ann Arbor, MI 48109 USA

**Keywords:** Retinal diseases, Macular degeneration, Biomedical engineering, Induced pluripotent stem cells, Imaging techniques and agents

## Abstract

Retinal pigment epithelial (RPE) cells play a crucial role in the health of the retina, and their dysfunction is associated with various ocular diseases. The transplantation of RPE cells has been proposed as a potential treatment for numerous degenerative diseases, including geographic atrophy from macular degeneration. However, current models to induce RPE damage in animal models prior to transplantation involve mechanical scraping, chemical administration, or laser photocoagulation techniques, which can damage the overlying neurosensory retina. This study aims to investigate the feasibility and efficacy of nanosecond duration laser treatment to safely remove large areas of RPE cells without causing damage to the adjacent tissue or affecting the retinal architecture. Twelve pigmented rabbits were treated with a nanosecond laser on each eye at a laser energy ranging from 200 to 800 nJ with a treated area of 5 × 5 mm^2^. Human induced pluripotent stem cells-differentiated to RPE (hiPSC-RPE) cells labeled with indocyanine green (ICG), an FDA approved dye, were transplanted subretinally into the damaged RPE areas at day 14 post-laser treatment. The RPE atrophy and hiPSC-RPE cell survival was evaluated and monitored over a period of 14 days using color photography, fluorescein angiography (FA), photoacoustic microscopy (PAM), and optical coherence tomography (OCT) imaging. All treated eyes demonstrated focal RPE loss with a success rate of 100%. The injured RPE layers and the transplanted hiPSC-RPE cells were visualized in three dimensions using PAM and OCT. By performing PAM at an optical wavelength of 700 nm, the location of hiPSC-RPE cells were identified and distinguished from the surrounding RPE cells, and the induced PA signal increased up to 18 times. Immunohistochemistry results confirmed the grafted hiPSC-RPE replaced regions of RPE damage. This novel technique has the potential to serve as an animal model of RPE degeneration, to improve models of RPE transplantation, and may help accelerate translation of this therapeutic strategy for clinical use.

## Introduction

Stem cell therapy (SCT) and regenerative medicine are demonstrating significant promise to improve treatment outcomes for various diseases, including diabetic retinopathy^1–3^, degenerative retinopathies including retinitis pigmentosa^4,5^, and geographic atrophy (GA) from dry age-related macular degeneration (AMD)^6–9^. Recently, several clinical trials have performed SCT to treat GA from AMD^7,9^. These treatments show great potential for patients by replacing the degenerated retinal pigment epithelium (RPE) cells. However, many factors remain to be elucidated, such as the fate, differentiation, and migration pattern of stem cells after transplantation^10^. Therefore, comprehensive evaluations should be further investigated to better understand the behavior of stem cells after transplantation. There were two major methods to transplant stem cells into the location of the targeted tissues: transplantation of stem cell–derived RPE (SC-RPE) monolayer sheets^11^ and suspension of SC-RPE cells^12^. Animal models to optimize cell therapy require removal of host RPE cells using a chemical procedure such as subretinal administration of sodium iodate and ethylenediaminetetraacetic acid^12–14^ or surgical tools (i.e., hydraulic debridement). Although these methods improve the integration of the transplanted SC-RPE, they can cause several severe side effects, including retinal toxicity and proliferative vitreoretinopathy, which can interfere with transplanted cell viability and function and therefore confound results. Therefore, it is essential to investigate alternative approaches that can non-invasively and safely remove RPE cells.

Several noninvasive methods to induce RPE degeneration have been investigated by using laser illumination. This method was first developed and described by Roider et al*.*^15^. The authors applied a laser-induced microbubble cavitation effect to disrupt the RPE cell wall^16,18^. In previous studies, our group has reported non-invasive and non-chemical methods to induce RPE damage in living rabbit models for progenitor cell transplantation using laser photocoagulation^17,18^. Although the RPE cell layer was injured after laser irradiation, we also found that the millisecond incident laser caused severe damage to other retinal layers in the treated areas, including the outer nuclear layer (*ONL*), outer plexiform layer (*OPL*), inner nuclear layer (*INL*), and ganglion cell layer (*GCL*). These layers were damaged or absent over the laser lesions.

To reduce or avoid collateral retinal damage to the neurosensory tissue, several recent research studies have focused on reducing the laser energy by increasing the laser wavelength or using either a shorter-duration pulse or noncontinuous wave micro-pulses^19–24^. The use of laser technology enables a targeted method for eliminating the retinal pigment epithelium (RPE) monolayer, typically around 10 µm in thickness^25,26^. Wood et al*.* have introduced an efficient method to ablate RPE cells using retinal regeneration therapy (2RT) system^23,24,27^. The advantage of this system is that it induces minimal collateral damage to photoreceptors at clinically relevant energy settings^28^. In addition, the 2RT laser has already been tested for the treatment of diabetic macular edema (DME) patients in the clinic and has shown to have an effect on retinal thickness reduction^29,30^.

Recently, microsecond duration laser has been applied to remove a large area of RPE in mice^31^ and rabbits^32,33^. The advantage of this method is its ability to avoid damaging the choriocapillaris and neurosensory retina, preserving the overlying retinal tissues. This method did not cause severe damage to the adjacent retinal tissues. These studies had some limitations, including short-term evaluation and lack of data on histological and immunohistochemical analysis. The absence of such data makes it difficult to understand the precise changes and mechanisms triggered by the laser treatment.

This study presents a novel method for selective large-area RPE removal for cell therapy animal models using a nanosecond pulse duration laser. During the laser irradiation, real-time optical coherence tomography (OCT) and CCD camera imaging-guidance systems were integrated to monitor the treatment. The local RPE degeneration areas were followed longitudinally over a period of 14 days using multimodal imaging, including color fundus photography, red-free photography, fundus autofluorescence (FAF), fluorescein angiography (FA), indocyanine green angiography (ICGA), and OCT. On day 14 after laser treatment, subretinal injection of human induced pluripotent stem cells-differentiated to RPE (hiPSC-RPE) labeled with ICG were administered to the rabbits with the RPE damage model to demonstrate the feasibility of this RPE damage animal model for regenerative cell therapy applications. The viability of the transplanted hiPSC-RPE cells was tracked over a period of 14 days using color fundus photography, FAF, ICG, FA, OCT, and photoacoustic microscopy (PAM) imaging. Histology and immunohistochemistry were performed to evaluate the dynamic change of retinal architecture, RPE degeneration, and the growth of the transplanted stem cells in the subretinal space.

## Results

### Longitudinal visualization of RPE after laser treatment

**Figure 1:**
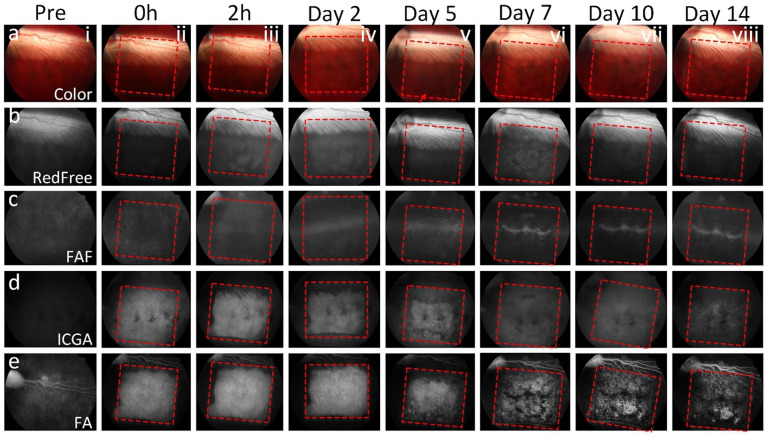
In vivo multimodal imaging visualization of RPE degeneration at 400 nJ. (**a**) Color fundus photographs obtained at different time points: pre- and post-laser irradiation at 400 nJ. Red dotted squares and arrows indicate the area of laser treatment. (**b**) Red free fundus images. (**c**) Fundus autofluorescence (FAF) images showing the degeneration of RPE cells over time after laser treatment. (**d**) Indocyanine green angiography (ICGA) images. Sharply demarcated hyperfluorescent areas (window defects) demonstrate evidence of RPE death. (**e**) Fluorescein angiography (FA) images. These FA images demonstrate sharply demarcated hyperfluorescence transmission (window) defects in areas with absent/dead RPE.

The fundus of all treated eyes was monitored before and after laser treatment at different time points: 10 min, 2 h, day 2, 5, 7, 10, and 14 using multimodal imaging including color fundus photography, red-free, FAF, ICGA, FA and SD-OCT. Before laser treatment, the morphology of retinal vessels and retinal structures like the optic nerve, nerve fiber layer (NFL), and RPE layer was clearly observed on color fundus photography (Fig. 1ai), red-free (Fig. 1bi), and FA images (Figs. 1ei, 2ai–bi). No evidence of abnormal signal was found on the FAF (Fig. 1ci) and ICGA (Figs. 1ci, 2ci–di). Ten minutes to 2 h after laser treatment at 400 nJ, there was no evidence of fundus and RPE cells affected by laser irradiation observed on the color image (Fig. 1aii–aiii). The laser-treated area was barely discernible on red-free and FAF (Fig. 1bii–biii,cii–ciii). In contrast, the laser treatment pattern was clearly observed on ICGA (Figs. 1dii–diii, 2cii–diii) and FA (Figs. 1eii–eiii, 2aii–biii) with sharply demarcated hyperfluorescence from transmission (window) defects from the degenerated RPE as described previously^34^. From day 2 to day 14, the laser-treated areas were clearly detected on all imaging modalities (Fig. 1aiv–aviii,biv–bviii,civ–cviii,div–dviii,eiv–eviii). It was noted that the death of RPE cells was distinctly visible from day 7 post-treatment with strong hyperfluorescence (Fig. 1evi–eviii).

**Figure 2:**
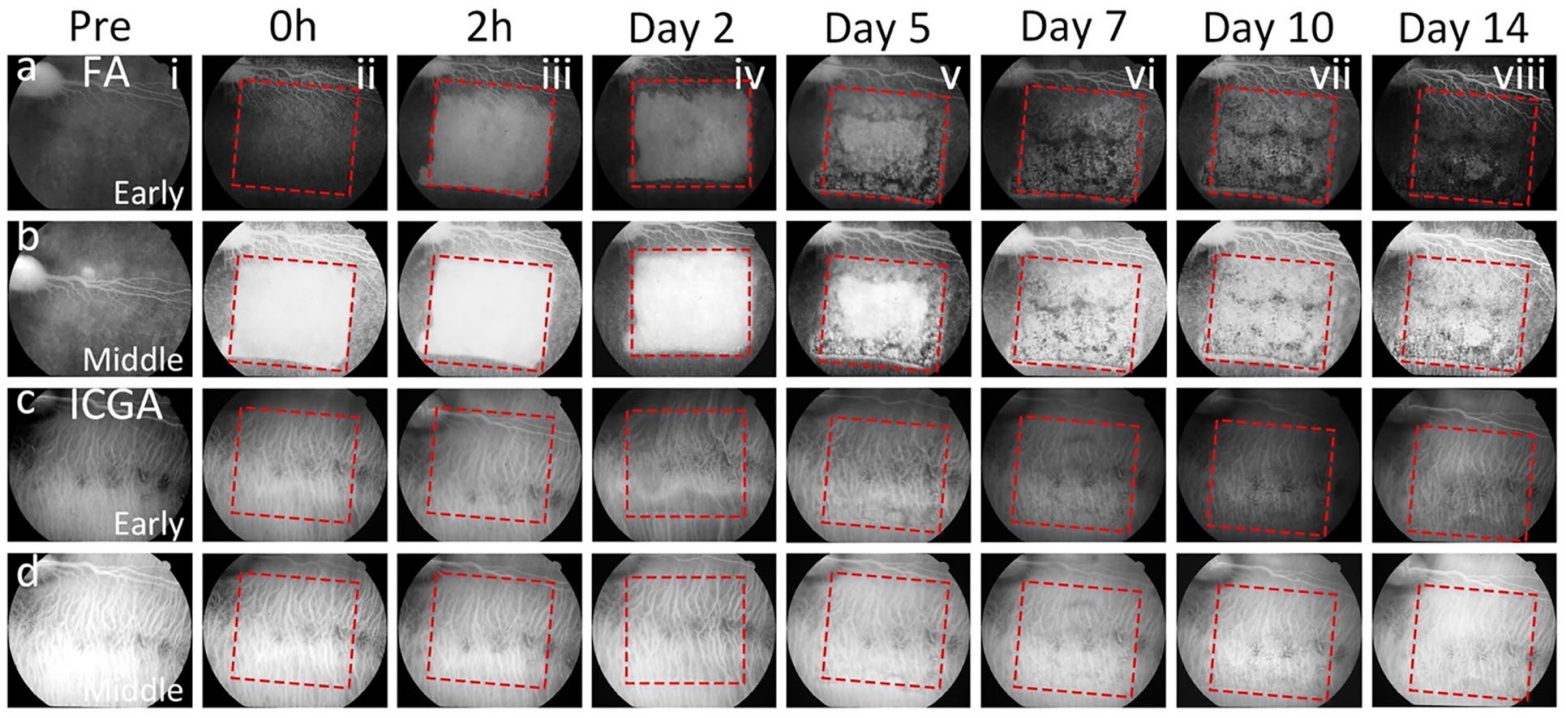
In vivo fluorescein angiography (FA) and indocyanine green angiography (ICGA) images of the retina. (**a–b**) Early and middle phase FA images captured at various intervals within a period of 14 days. Initially, both retinal vessels and the retinal pigment epithelium (RPE) layer exhibit normal features prior to treatment (**ai, bi**). Post-treatment, while retinal vessels remain unaffected by laser illumination, significant alterations are observed in the RPE layer, clearly discernible through treatment patterns outlined by red dotted squares (**aii–aviii, bii–bviii**). (**c–d**) Early and middle phase ICGA images taken before and after treatment at different time points. No discernible changes in choroidal morphology were evident before and immediately after treatment at 0 h and 2 h (**ci–ciii, di–diii**). However, treatment patterns marked by red dotted squares become apparent in ICGA images from day 2 to day 14, indicating the progression of treatment effects (**civ–cviii, div–dviii**).

**Figure 3:**
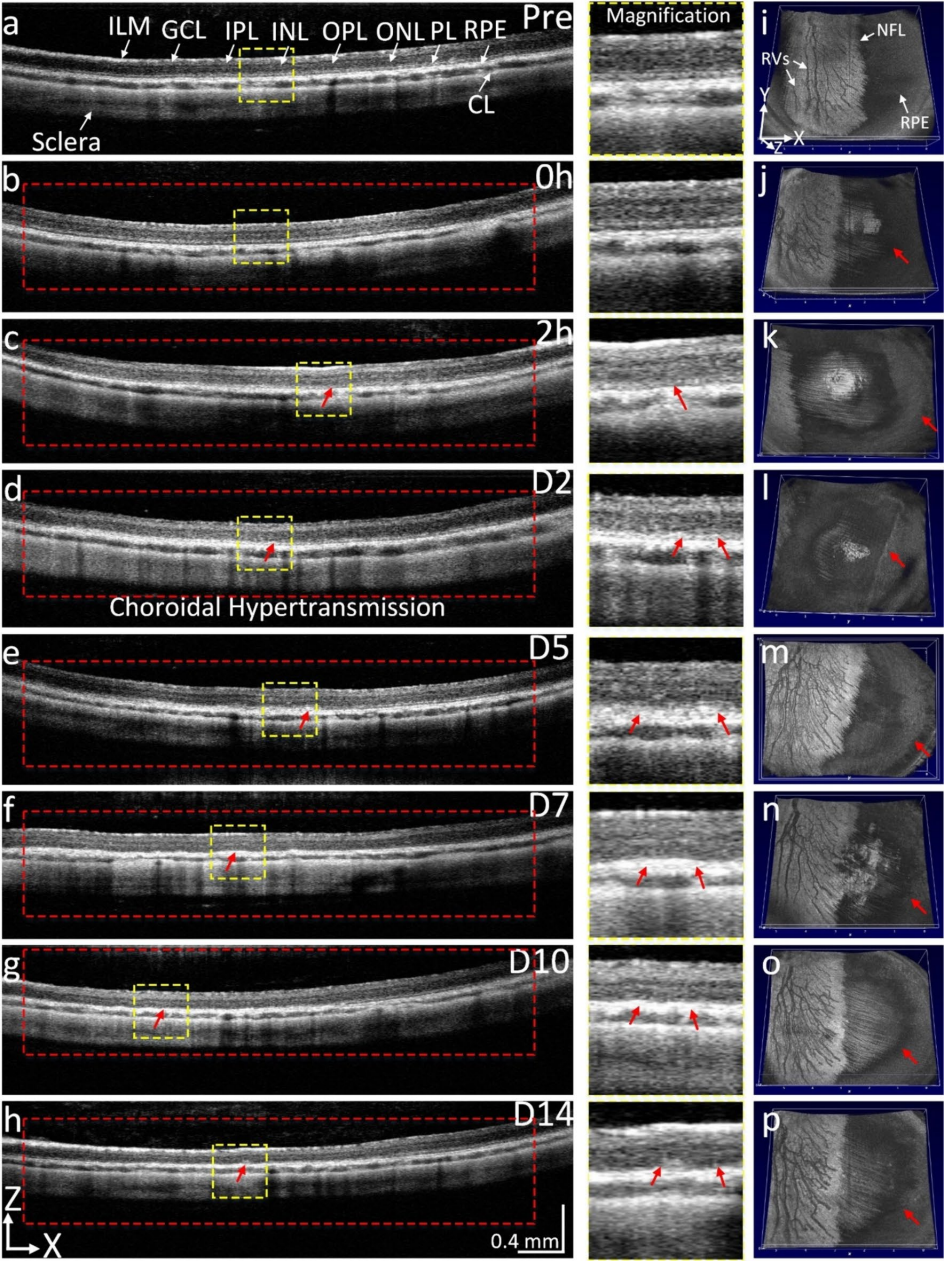
OCT tracking RPE degeneration at 400 nJ. (**a**) 2D OCT image acquired before laser treatment showing the different retinal layers, including inner limiting membrane (*ILM*), ganglion cell layer (*GCL*), inner plexiform layer (*IPL*), inner nuclear layer (*INL*), outer nuclear layer (*ONL*), outer plexiform layer (*OPL*), photoreceptors layer (*PL*), RPE, choroid layer (*CL*), and sclera. (**b–h**) B-scan OCT images obtained immediately (0 h), 2 h, days 2, 5, 7, 10, and 14 post-laser illumination at 400 nJ. Red rectangles show the treated areas. Choroidal hypertransmission was observed from immediately post treatment up to day 14 (red arrows). Magnification of choroidal hypertransmission regions were extracted from dotted yellow squares display in Figures **a–h**. (**i–p**) 3D OCT images showing entire structure of the retina including retinal vessels, nerve fiber layer (*NFL*), and RPE. No evidence of neurosensory retinal tissue changes pre- and post-treatment.

To visualize the dynamic change of retina architecture, we performed SD-OCT imaging before and after treatment at different time points. The B-scan OCT images acquired before treatment show different retinal layers including ILM, IPL, INL, OPL, ONL, ellipsoid zone, RPE, choroid, and sclera (Fig. 3a). The corresponding 2D OCT images obtained at 15 min and 2 h post-laser treatment show normal retinal structure without damage to the outer retinal layers (Fig. 3b,c). No retinal detachment was found on the OCT images. We found that strong hyper-reflectivity was detected at the level of the choroid and sclera within the treated area (red dotted rectangle) starting on day 2 post-treatment, indicating RPE atrophy and choroidal hypertransmission (Fig. 3d and magnification images (middle column)). The treated RPE cells were clearly observed up to 14 days (Fig. 3e–h) while the other retinal layers remain normal. *En face* 3D OCT images show the entire structure of the retina. No evidence of morphological changes was observed on the retinal vessels, and NFL before and after treatment (Fig. 3i–p).

### Laser fluence for RPE cell damage

**Figure 4:**
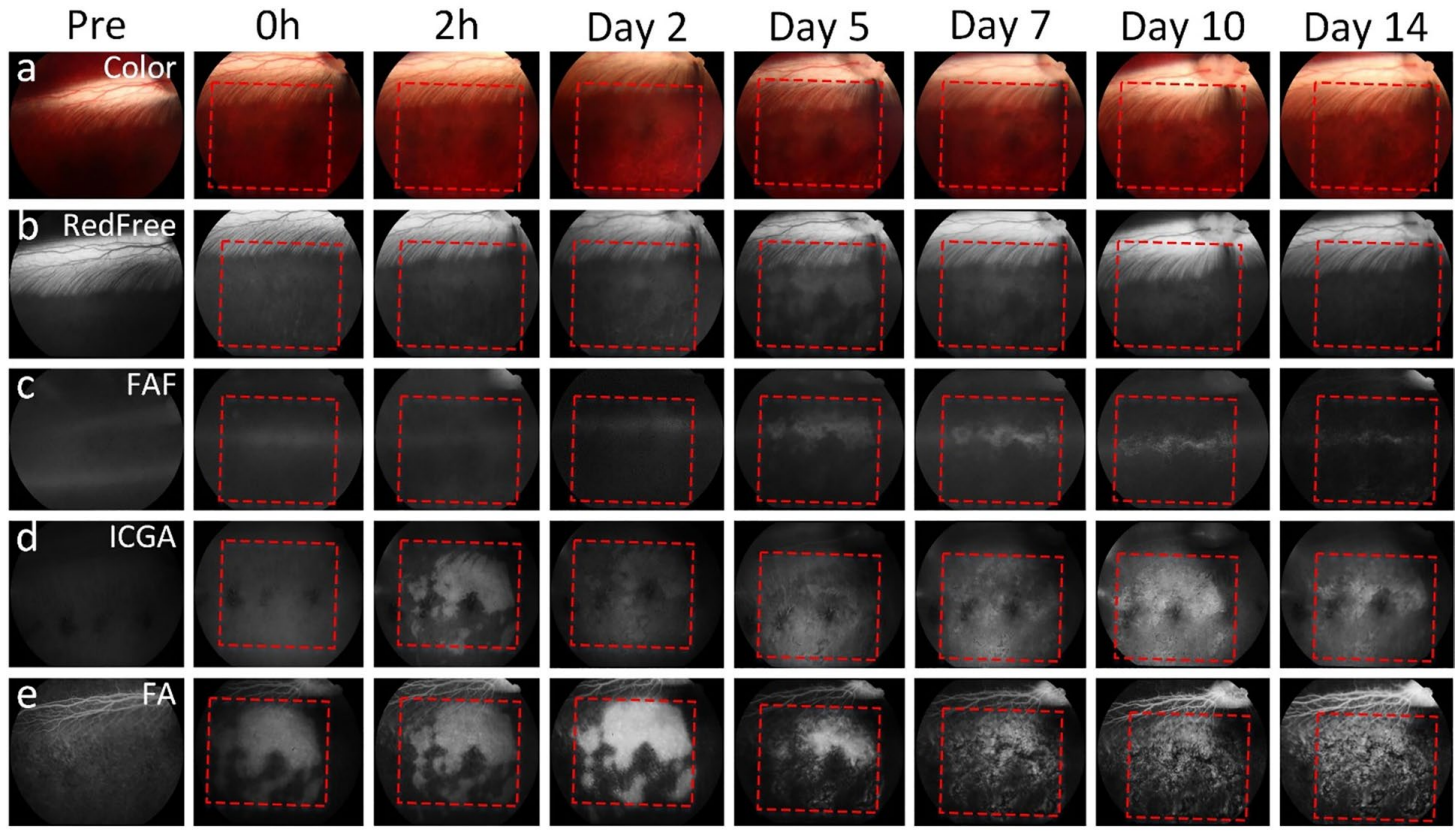
In vivo multimodal imaging visualization of RPE degeneration at 200 nJ. (**a**) Color fundus photographs obtained at different time points pre- and post- laser irradiation at 200 nJ. Red dotted squares and arrows indicate the area of laser treatment. (**b**) Red free fundus images. (**c**) Fundus autofluorescence (*FAF*) images showing the degeneration of RPE cells over time after laser treatment. (**d**) Indocyanine green angiography (*ICGA*) images. Sharply demarcated hyperfluorescent areas (window defects) demonstrate evidence of RPE cell loss. (**e**) Fluorescein angiography (*FA*) images. These FA images demonstrate sharply demarcated hyperfluorescence transmission (window) defects in areas with absent/dead RPE.

**Figure 5:**
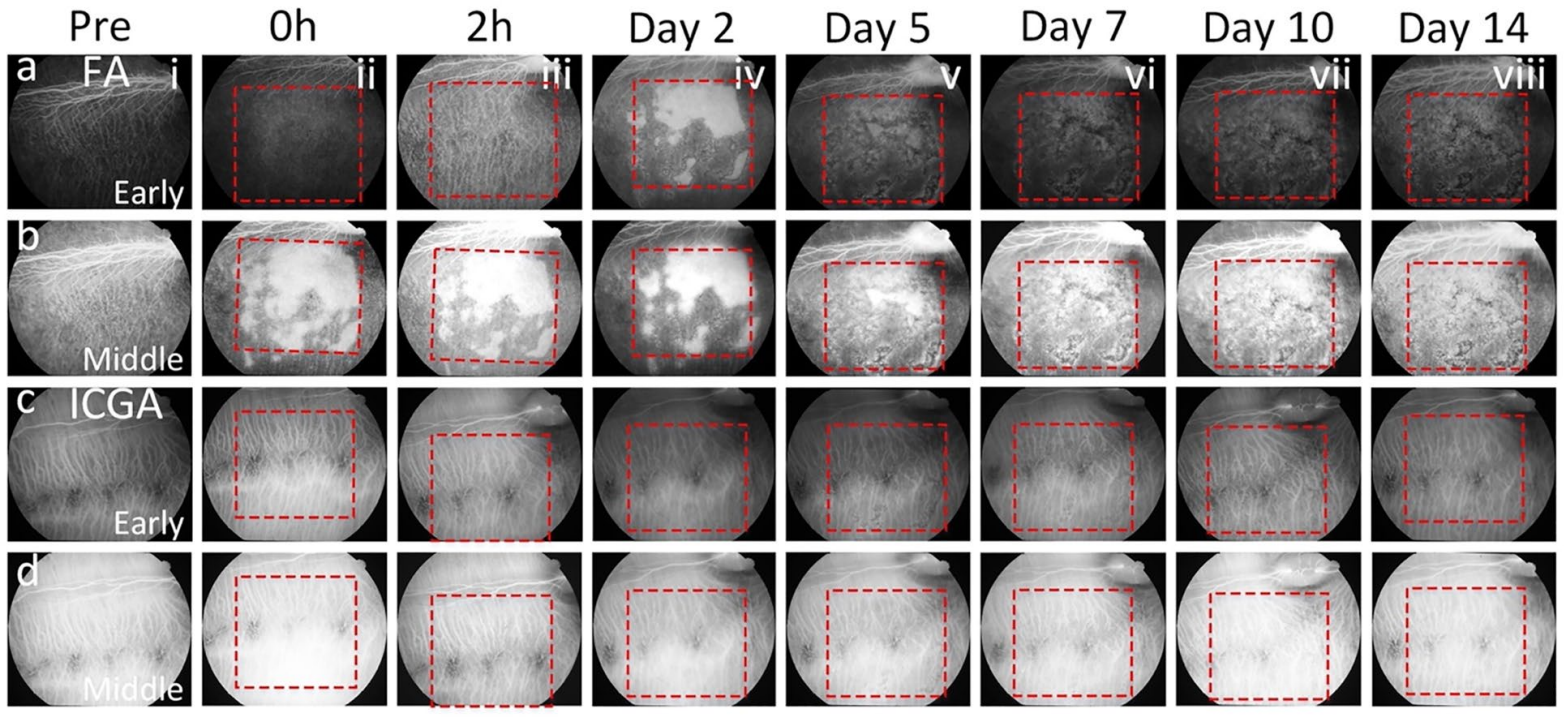
In vivo images of the retina were obtained using fluorescein angiography (*FA*) and indocyanine green angiography (*ICGA*). (**a–b**) display early and middle phase FA images taken over a period of 14 days. Both the retinal vessels and RPE layer appear normal before treatment (**ai, bi**). Following treatment, while the retinal vessels remain unaffected by laser exposure, noticeable changes occur in the RPE layer, highlighted by treatment patterns outlined with red dotted squares (**aii–aviii, bii–bviii**). (**c–d**) show early and middle phase ICGA images captured before and after treatment at different time points. There were no observable changes in choroidal morphology before and immediately after treatment at 0 and 2 h (**ci–ciii, di–diii**). However, treatment patterns, indicated by red dotted squares, become evident in ICGA images from day 2 to day 14, signifying the progression of treatment effects (**civ–cviii, div–dviii**).

To determine the optimal of laser fluence for RPE removal, we implemented two further experiments using laser fluences at 200 and 800 nJ. The treated area was about 5 × 5 mm for the 200 nJ group and 3 × 3 mm for the 800 nJ group (Figs. 4, 5 and Supplementary Fig. S1). We found that the RPE affected by laser illumination at 200 nJ could only be observed by late phase FA images while color fundus, red-free, FAF, and late phase ICGA did not show any evidence of RPE atrophy at 15 min post-treatment. The RPE affected by laser treatment was clearly observed at 2 h post-treatment. We found that the RPE cells affected by laser treatment were not homogenous. There are some portions of RPE within the treated areas that were not affected after laser irradiation. This might be explained by the nonuniformity and significant variation of melanin within RPE cells. When the laser illuminated the sample, the absorption of laser in regions of low melanin expression was lower than in another area with higher melanin expression. Thus, the local laser treatment effect might not have been enough to induce RPE cells death or necrosis. However, choroidal hypertransmission area with a transmission (window) defect was clearly observed from day 5 to day 14 post-treatment with strong hyperfluorescence. When the animals were treated with 800 nJ, the treatment patterns were observed on all imaging modalities (fundus, red free, FAF, ICGA, and FA) immediately post-treatment (0 h). More hyperfluorescence was observed in the treated areas from day 5 post-laser treatment (Supplementary Fig. S1).

**Figure 6:**
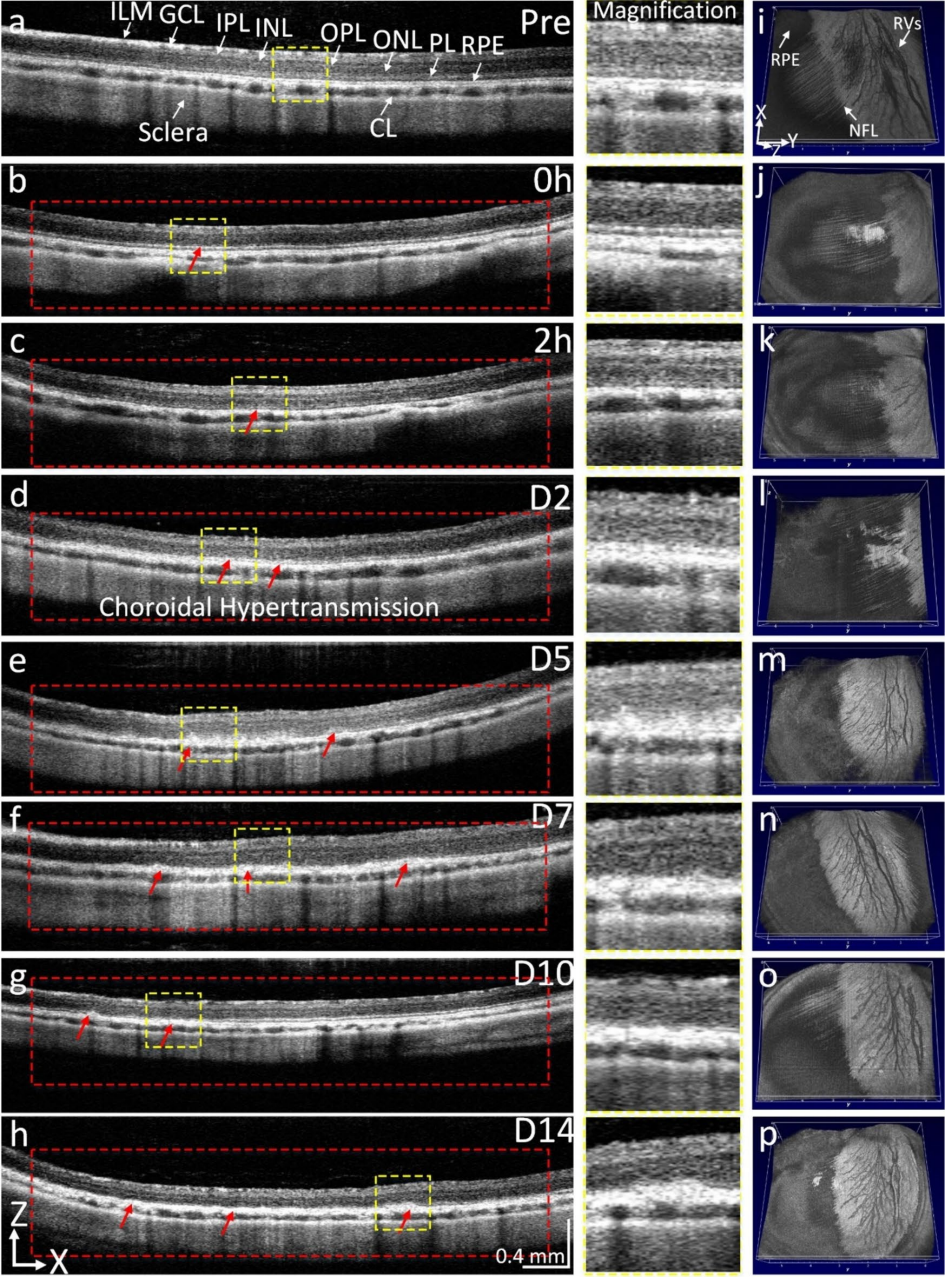
OCT imaging after laser treatment at 200 nJ. (**a**) B-scan OCT images obtained pre laser irradiation showing different retinal layers, including inner limiting membrane (*ILM*), ganglion cell layer (*GCL*), inner plexiform layer (*IPL*), inner nuclear layer (*INL*), outer nuclear layer (*ONL*), photoreceptors layer (*PL*), RPE, choroid layer (*CL*), and sclera. (**b–h**) B-scan OCT images obtained immediately (0 h), 2 h, days 2, 5, 7, 10, and 14 post-laser illumination at 200 nJ. Red rectangles show the treated areas. Choroidal hypertransmission (red arrows) was observed from days 2 to day 14 post-treatment. Magnification images show the isolated choroidal hypertransmission areas extracted from dotted yellow squares display in Figures **a–h**. (**i–p**) 3D OCT images showing entire structure of the retina including retinal vessels, nerve fiber layer (NFL), and RPE. The RPE layer was changed post-treatment demonstrating choroidal hypertransmission.

The OCT images also demonstrate that RPE cells were significantly affected by laser treated at 200 nJ with choroidal and scleral hypertransmission within the treated areas (Fig. 6). There was no retinal detachment observed in the treated animals. There was no significant damage noted to the neurosensory retinal layers such as the ILM, INL, and ONL.

### Retinal thickness measurement

**Figure 7:**
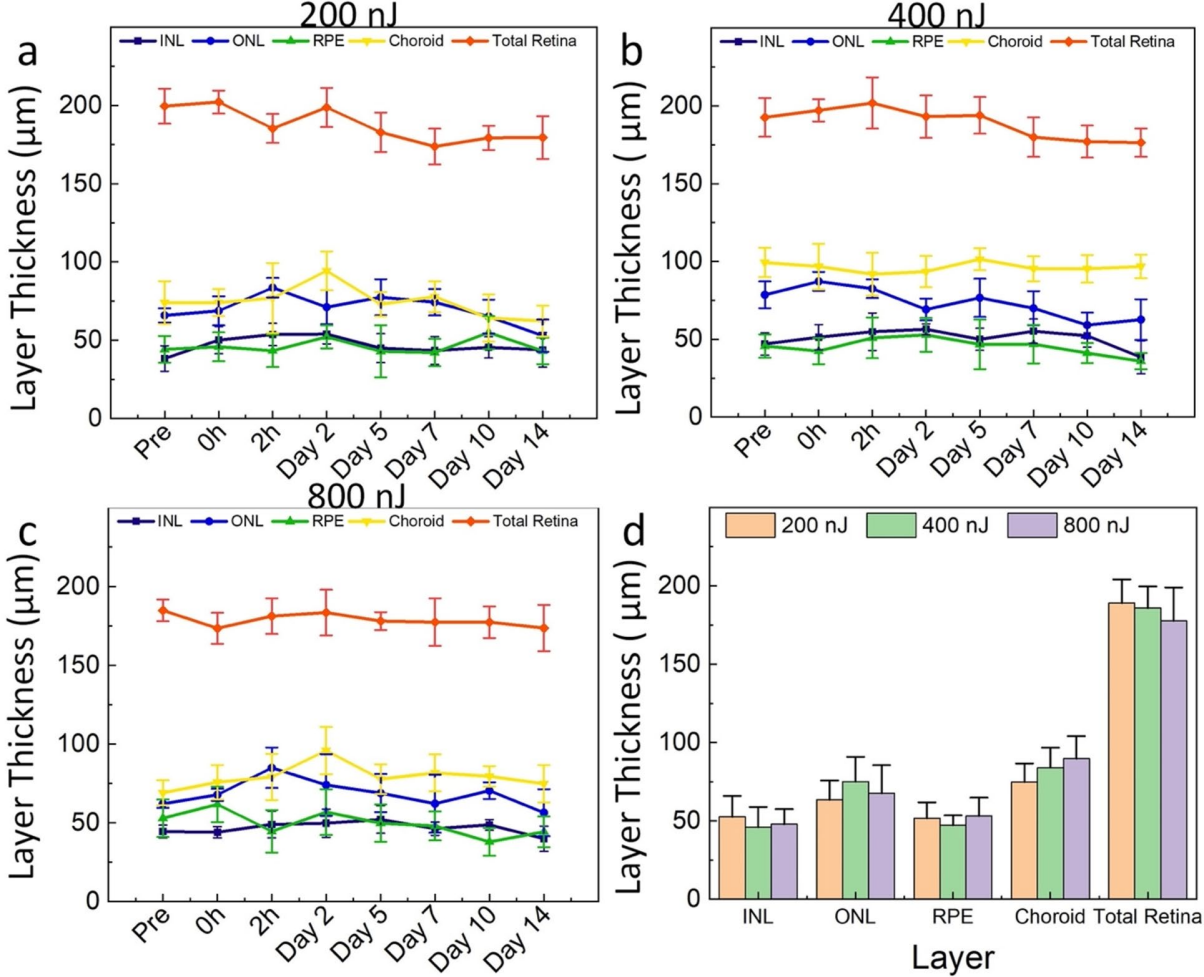
Quantitative measurement of the retinal thickness: (**a**) Quantification of thickness of different retinal layers before and after laser treatment at 200 nJ including INL, ONL, RPE, choroid, and total retinal thickness. (**b–c**) Graph of retinal thickness obtained after laser treatment at 400 and 800 nJ, respectively. (**d**) Comparison of different retinal layers under laser illumination at different laser energies of 200, 400, and 800 nJ at day 14 (n = 3, p > 0.05).

To evaluate the potential side effects of nanosecond laser irradiation to the retina, retinal thickness measurements were performed using B-scan OCT images. Different retinal layers were determined including INL, ONL, RPE, choroid, and total retina thickness. The INL, ONL, RPE, and choroid layers were segmented. The thickness was measured using ImageJ software. The results are displayed in Fig. 7. The quantitative retinal thickness measurement illustrates that there were no significant changes observed in the INL, ONL, RPE, choroid, and total retinal thickness after laser treatment at 200 nJ (Fig. 7a), 400 nJ (Fig. 7b), or 800 nJ (Fig. 7c) in comparison to before laser treatment. Total retinal thickness changed less than 12% at day 14 post-laser treatment at 200 nJ (thickness = 239.4 ± 13.38 µm for pre-treatment and 213.06 ± 15.06 µm for post-treatment). Similarly, the total retinal thickness was slightly changed about 10.9% for post-treatment at a laser energy of 400 nJ (thickness = 215.73 ± 13.81 µm post-treatment). When compared with laser treatment at various energies of 200, 400, and 800 nJ, the INL thickness was about 3.6–13.9% different between these treatment groups (Fig. 7d). Similarly, the ONL thickness fluctuated around 6.2 to 15.5%, RPE layer about 2.5 to 11%, and choroidal layer about 6.3 to 16.6% between these treatment groups.

### Stem cell transplantation and visualization

**Figure 8:**
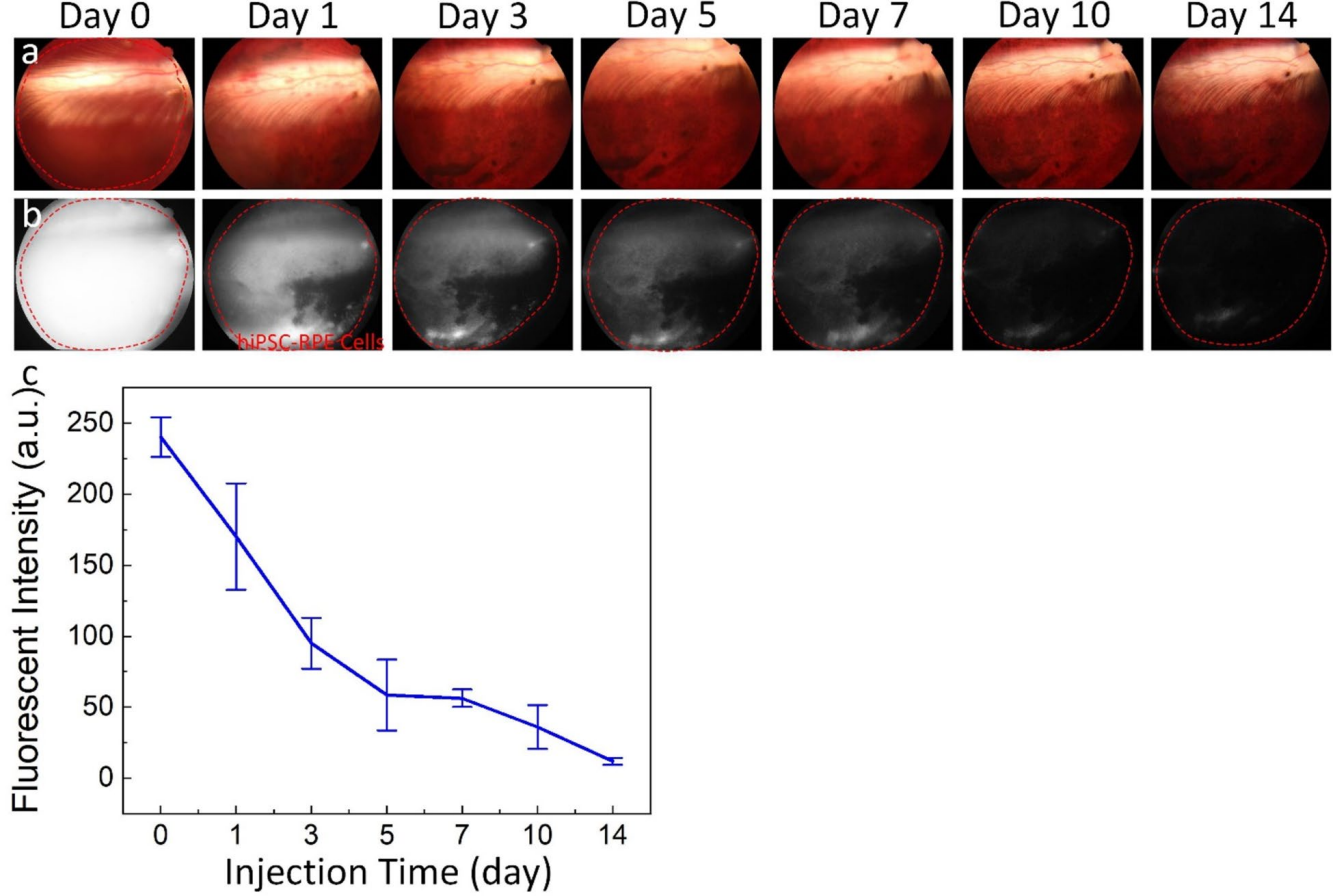
In vivo imaging-guided stem cell transplantation. (**a**) Color fundus photographs showing the location of the injection subretinal bleb immediately after transplantation of 10^5^ hiPSC-RPE cells labeled with ICG dye and the distribution of hiPSC-RPE cells over time. (**b**) Corresponding fluorescent images obtained at the same location shown in Figure **a**. Red dotted circles indicate the distribution and migration of the transplanted hiPSC-RPE cells over time. (**c**) Quantitative fluorescent intensity measured as a function of time post-injection. Data shown as mean and standard deviation (n = 3, p < 0.01).

To demonstrate the ability of stem cells to replace damaged RPE caused by nanosecond laser, we performed subretinal transplantation of hiPSC-RPE cells labeled with ICG dye conjugated with RGD peptides. Figure 8 shows the distribution of the transplanted hiPSC-RPE cells obtained by color fundus photography (Fig. 8a) and fluorescent imaging (Fig. 8b) pre-injection, immediately post-injection, and longitudinally for up to 14 days of follow-up. It was difficult to visualize the distribution of the transplanted cells with color fundus images due to the similar appearance of the hiPSC-RPE cells and native RPE cells. In contrast, the grafted cells were clearly observed using ICG fluorescent imaging at day 0 and were detectable up to day 7 after the injection. The injection blebs covered almost the entire RPE damaged areas. This allows the transplanted cells within the damaged areas to replace the damaged RPE. A less bright fluorescent signal was observed on day 11 and day 14 post-injection, possibly due to the degeneration of ICG dye in the bioenvironment. The fluorescent intensity (FI) extracted from the fluorescent images was determined using the region of interest (ROI) method. The quantification results demonstrate that the FI gradually decreased over time and was significantly reduced by day 14 post-injection (Fig. 8c). The FI reached the peak at day 0 post-injection and exhibited about 67.5-fold higher signal than that of background (FI = 240.36 ± 13.85 (a.u.) for immediately post-injection vs 3.56 ± 1.21 (a.u.) for pre-injection). Then, the fluorescent signal gradually reduced over time but still achieved about 3.3-fold higher signal when compared to the background at day 14 (FI = 11.78 ± 2.51 (a.u.) for post-injection at day 14).

**Figure 9:**
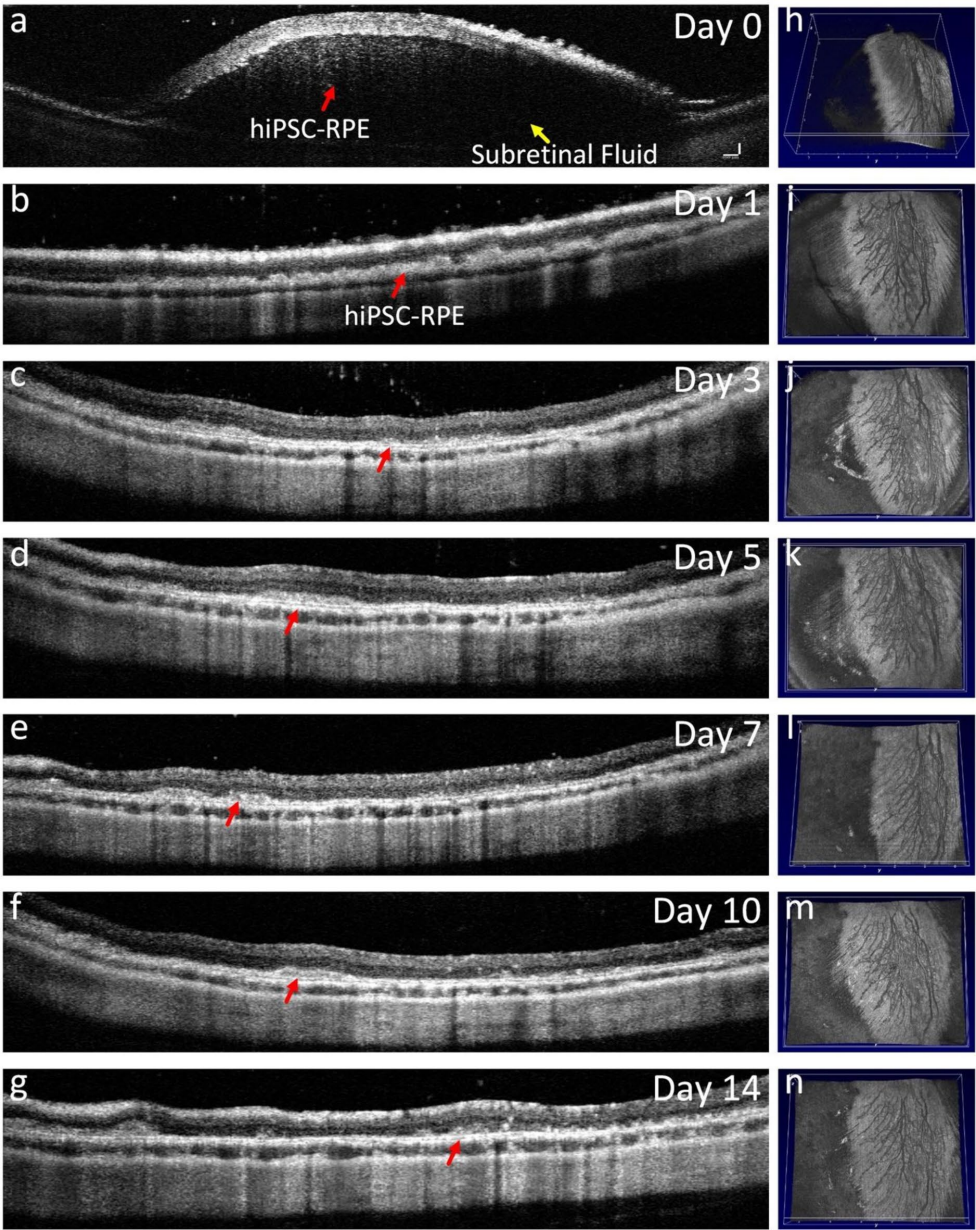
In vivo OCT image of stem cells after transplantation. (**a**) OCT images acquired immediately after the hiPSC-RPE cells were delivered into the subretinal space through subretinal injection. The labeled hiPSC-RPE cells were clearly observed in the subretinal space along with the accumulation of subretinal fluid (yellow arrow). (**b-g**) 2D OCT images obtained at different time points post-transplantation. The location of the transplanted hiPSC-RPE cells were clearly detected at the RPE layer with high contrast. (**h-n**) 3D OCT images.

Figure 9 exhibits the 2D and 3D OCT images of the transplanted hiPSC-RPE cells at different time points: 0, 1, 3, 5, 7, 10 and 14 days. At day 0, the images show retinal detachment with the presence of subretinal fluid, and the labeled hiPSC-RPE cells are observed in the subretinal space (Fig. 9a). Subretinal fluid was completely resolved on day 1, while hyper-reflective subretinal material remained, suggesting that the transplanted cells remained in the subretinal space (Fig. 9b). We found that the cells that were transplanted into the retina gradually integrated into the damaged tissue over a period from days 3 to 14 with stronger signal than that of the adjacent tissues as indicated by red arrows (Fig. 9c–g). This data provided a visual representation of the location of the transplanted stem cells, allowing for a better understanding of the success and progress of the regenerative tissues. The replacement of damaged RPE cells by the transplanted stem cells suggests potential for successful engraftment. Figure 9h–n show 3D OCT scans acquired at different time points. These images capture the changes in the retinal morphology and the integration of the transplanted stem cells into the subretinal space over time.

**Figure 10:**
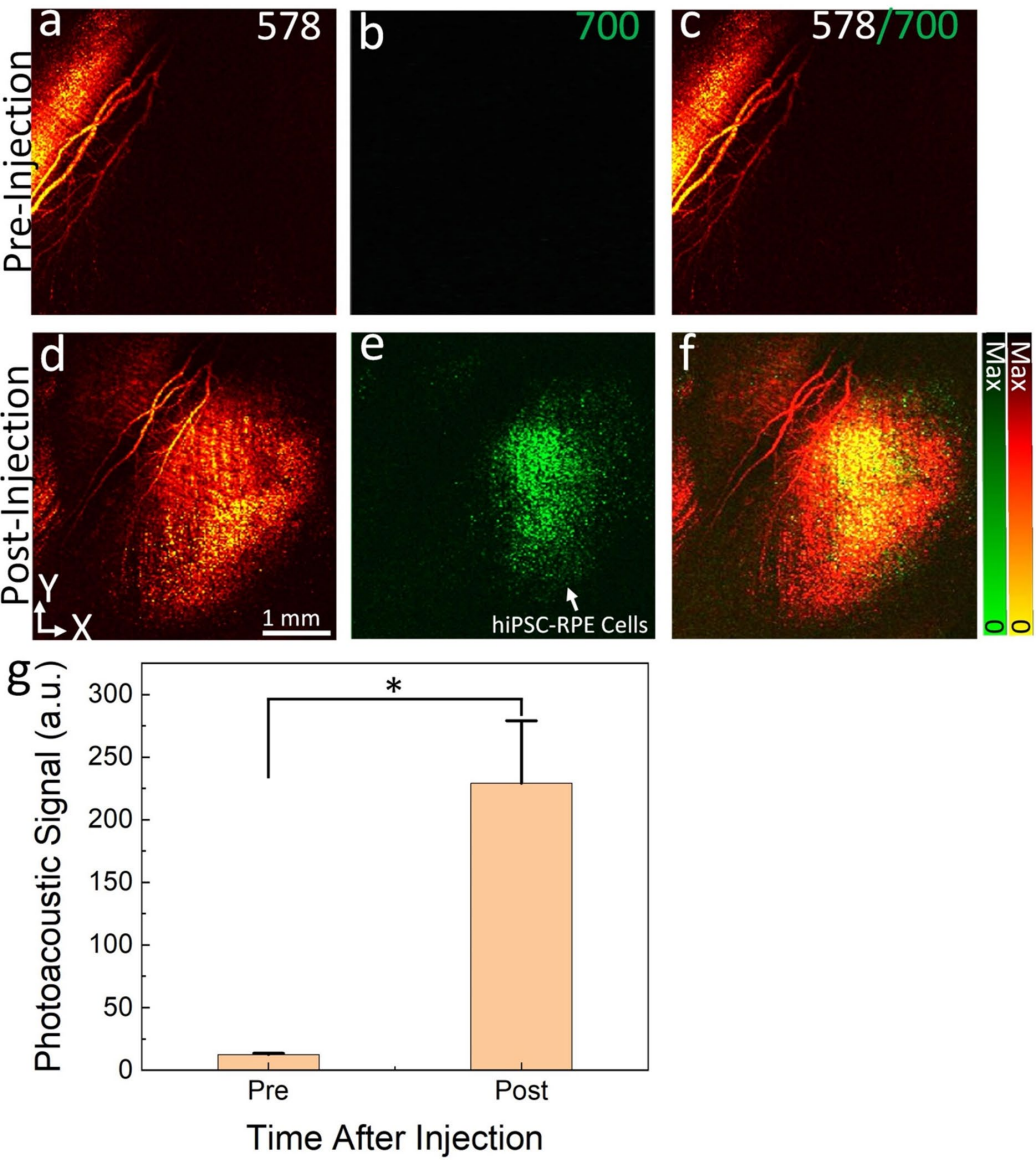
In vivo PAM imaging visualization the distribution of stem cells. (**a-b**) PAM images obtained at two different wavelengths of 578 nm (**a**) and 700 nm (**b**) at day 14 post-RPE removal. (**c**) Overlay PAM image obtained from **a** and **b**. (**e-d**) PAM images achieved at day 14 post-stem cells transplantation. Green color indicates the location of ICG in the hiPSC-RPE cells. (**f**) Overlay PAM images obtained from **d** and **e**. (**g**) Quantification PA signals (n = 3, p < 0.05).

To distinguish the transplanted cells from the native RPE cells, we performed PAM imaging at two time points: day 4 post-laser illumination (pre injection) and day 14 post-subretinal injection. Figure 10 presents the results of the PAM imaging, showing the differences between the transplanted cells and the native RPE cells. Figure 10a,d demonstrates the PAM images acquired at the excitation wavelength of 578 nm. These images show clearly the morphology of retinal vessels, capillaries, and melanin. The wavelength of 578 nm is chosen because it corresponds to the absorption peak of hemoglobin (Hb) in blood vessels and also includes melanin, which is a pigment found in the RPE and other tissues of the eye. Hb and melanin absorb light strongly at 578 nm, allowing for clear visualization of blood vessels and melanin-containing structures in the retina. The ability to clearly visualize the morphology of retinal vessels, capillaries, and melanin using PAM at 578 nm can provide valuable information for understanding retinal physiology, pathology, and potential changes associated with diseases or treatments. It can also aid in assessing the success of transplantation procedures, evaluating the health of the RPE, and investigating the effects of interventions on retinal structures. The PAM images obtained at 700 nm pre- and post-injection have revealed specific characteristics or markers from ICG dye that distinguish the transplanted cells from the native RPE cells (Fig. 10b,e). Minimal PA signal was observed on PAM obtained before the injection (Fig. 10b) due to low absorption of Hb at this wavelength. In contrast, strong PAM signal detected post cells injection (Fig. 10c) as a result of strong absorption laser light of the internalized ICG inside the cells. Figure 10c,f show the overlay PAM images obtained from two different wavelengths on the same orthogonal image planes. The distribution of the hiPSC-RPE cells were clearly distinguished from the surrounding retinal blood vessels and melanin (Fig. 10f). Figure 10g shows a graph of the quantitative PA signal measurements at a different location from the PAM images obtained at 700 nm. This illustrates that the photoacoustic (PA) signals showed a significant increase (18-fold) in the transplanted cells compared to the pre-injection baseline (PA_Signal_ = 229.02 ± 50.01 (a.u.) for post-injection vs 12.60 ± 0.87 (a.u.) for pre-injection). This finding suggests that the transplanted cells with ICG dye exhibit a higher photoacoustic response at 700 nm, indicating differences in their optical absorption properties compared to the native RPE cells or other surrounding tissues. The increased PA signals in the transplanted cells could be indicative of their survival and engraftment in the subretinal space, as well as their potential to function as intended. The enhanced signals may also suggest that the transplanted cells have successfully integrated into the host retina and are actively contributing to the retinal tissue’s photoacoustic properties.

### Histological analysis

**Figure 11:**
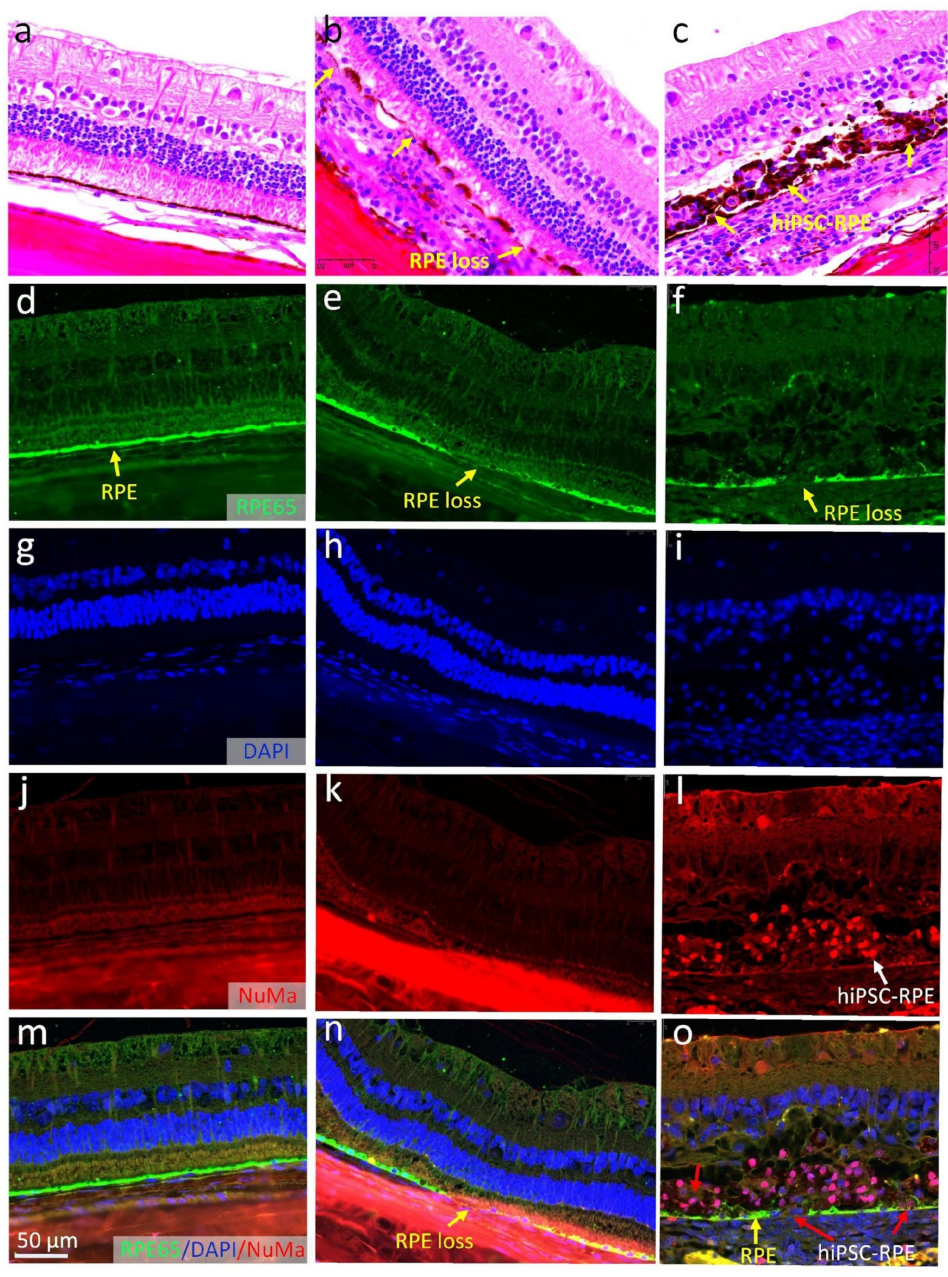
Histological and immunohistochemical analysis. (**a**) H&E staining image of control group without laser treatment showing the normal morphology of retinal cells and the architecture of retinal structure. All retinal tissues were clearly observed including ILM, GCL, IPL, INL, ONL, OPL, PL, RPE, choroid, and sclera. A single continuous RPE monolayer was detected. (**b**) H&E images without stem cells injection. RPE layers were significantly changed with discontinuities and regions of RPE loss were found in the RPE layer (yellow arrow). (**c**) H&E images of the tissues treated with laser followed by stem cell transplantation. Yellow arrows indicate the evidence of the transplanted hiPSC-RPE cells grafted in the subretinal space with high pigmentation (black color). (**d–o**) Immunofluorescent images of the tissues stained with two different antibodies: RPE65 and NuMa. RPE65-positive indicate the signal from RPE cells. Red fluorescent color shows NuMa positive hiPSC-RPE cells. Blue fluorescent color demonstrates the cell’s nuclei stained by DAPI. No NuMa-positive signal was detected on the control group and laser treatment group. RPE loss was detected on treated group (yellow arrows). The hiPSC-RPE cells replaced RPE death were observed on the stem cell transplantation groups (Figure **o**). Red arrows show the growing of hiPSC-RPE cells at the RPE damaged areas.

The treated animals in the study were euthanized to perform histological and immunohistochemical analyses to evaluate the potential of the transplanted human induced pluripotent stem cells differentiated to retinal pigment epithelium (hiPSC-RPE) for replacing damaged RPE cells caused by nanosecond laser illumination. The thin section of treated tissues was stained with H&E, RPE65, and NuMA antibodies to assess the survival, integration, and functional characteristics of the transplanted hiPSC-RPE cells in the retinal tissue following laser-induced RPE damage. Figure 11a illustrates the H&E image of the sample without being treated with the laser. The cells in this group appear normal, with well-defined cellular boundaries, intact cell nuclei, and cytoplasm that are uniformly stained with pink Eosin. The retinal tissue architecture shows no signs of cellular damage or abnormalities. Distinct retinal layers were observed clearly such as the internal limiting membrane (*ILM*), nerve fiber layer (*NFL*), ganglion cell layer (*GCL*), inner plexiform layer (*IPL*), inner nuclear layer (*INL*), outer plexiform layer (*OPL*), outer nuclear layer (*ONL*), external limiting membrane (*ELM*), photoreceptor (*P*), RPE, and choroid layer (*C*). A monolayer of RPE was detected with high melanin pigmentation. In Fig. 11b, the H&E image of the retinal tissues treated with laser shows noticeable changes specifically in the RPE layer, while the other retinal layers such as the ILM, GCL, IPL, INL, OPL, ONL, and other layers appear unchanged. There was noted to be no alterations in cellular morphology, such as disrupted cellular arrangement, irregular cell shape, or changes in staining characteristics compared to the control group. No retinal detachment was observed in the treated tissues. Figure 11c demonstrates the H&E image of the treated group followed by hiPSC-RPE cell transplantation and shows the mass of transplanted cells in the subretinal space.

Figure 11d–o shows representative immunostaining images using two different antibodies: anti-RPE65 specific to rabbit RPE65, and anti-NuMA specific to human NuMA, to distinguish rabbit and human RPE cells. The RPE65-positive cells were detected in the control group (Fig. 11d) and treated groups (Fig. 11e,f). But the absence of RPE65 at the focal site of laser treatment (Fig. 11e,f), confirmed that the laser treatment resulted in the removal or loss of RPE cells in the treated area. Figure 11j–l shows immunostaining with anti-NuMA antibody. The presence of NuMA-positive signals was observed in the treatment areas of the group that received hiPSC-RPE cell transplantation, but not in the native RPE cells from the control group (Fig. 11j) or the group without cell transplantation (Fig. 11k). This confirms the presence of hiPSC-RPE cells that were transplanted into the subretinal space. Figure 11m–o illustrates the overlay images, clearly showing the location of RPE loss and the distribution of hiPSC-RPE cells at the site of the laser lesion. Minor changes are noted in the photoreceptor layer and adjacent outer retinal layers (Fig. 11o).

**Figure 12:**
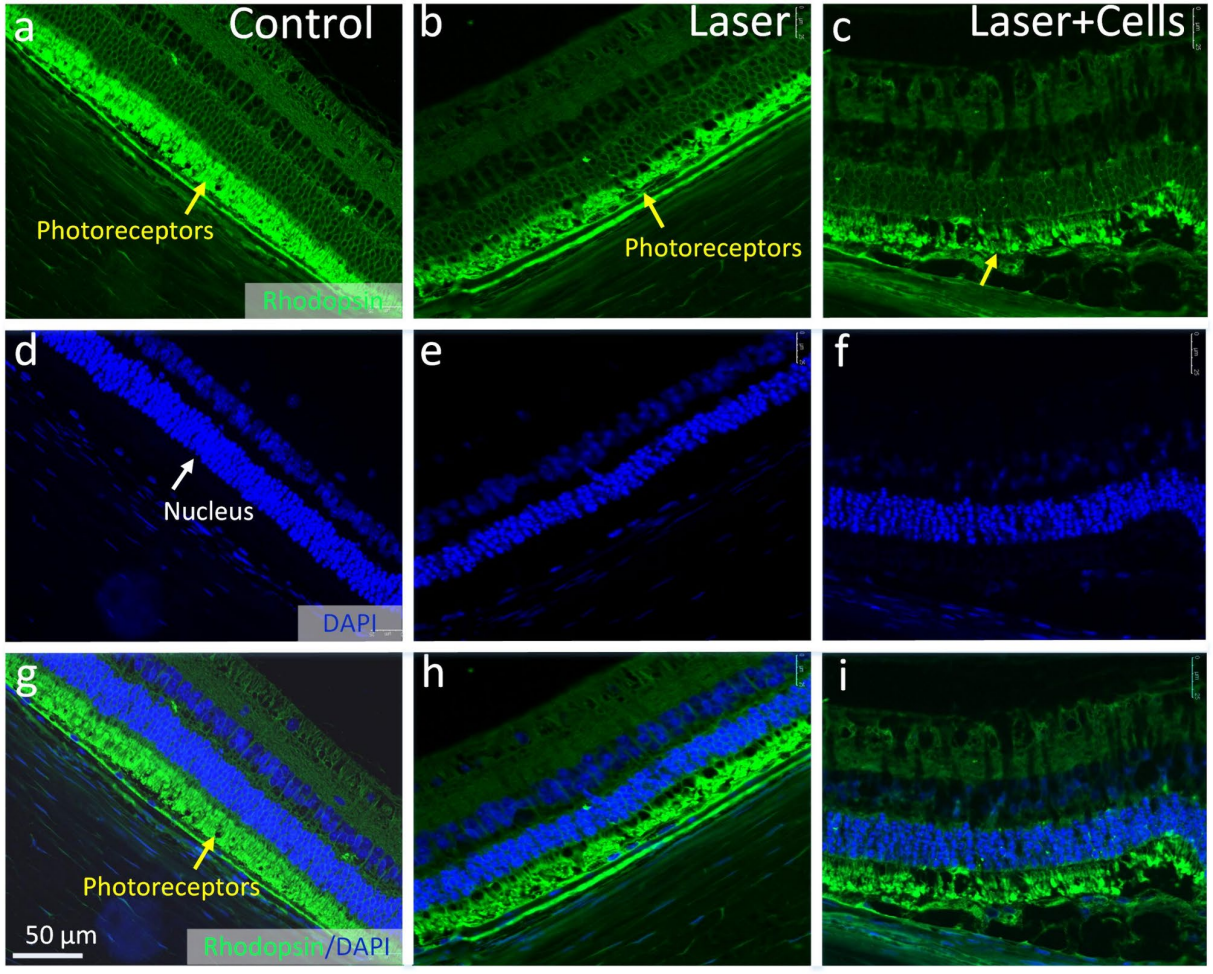
Immunofluorescence staining of Rhodopsin for photoreceptors. (**a, d, g**) Control group. (**b, e, h**) Treated group with laser only. (**c, f, i**) The treated group with laser followed by hiPSC-RPE cell transplantation. The green fluorescence color highlights the outer segment of photoreceptors stained with Rhodopsin, while cell nuclei are stained with DAPI in blue.

Figure 12 presents immunofluorescence staining images for photoreceptors utilizing the anti-rhodopsin antibody. The outer segments of photoreceptors (depicted in green) were distinctly identified in both the treated groups (Fig. 12b,c,h,i) and the control group (Fig. 12a,g). Interestingly, a mild de-structuring of photoreceptors was observed in the treated group with cell transplantation (Fig. 12c,i), indicated by a yellow arrow. Cell nuclei were stained by DAPI (Fig. 12d–f).

## Discussion

This study demonstrates a novel animal model of nanosecond laser-induced retinal pigment epithelium (RPE) removal for the study of different retinal degenerative diseases such as retinitis pigmentosa, macular dystrophies, and geographic atrophy from AMD. The nanosecond laser induced RPE removal model offers several advantages, including precision, reproducibility, non-invasiveness, versatility, and time efficiency, and translational relevance. This technique allows for precise and localized removal of RPE cells in a controlled manner. The laser can be precisely focused on the targeted RPE area, allowing for selective removal of RPE cells without damaging other retinal layers. This allows researchers to create well-defined experimental conditions and study the effects of RPE loss in a controlled manner. In addition, the nanosecond laser-induced RPE removal model offers reproducibility, as the laser parameters, such as laser power, duration, and spot size, can be standardized and controlled. This potentially allows for consistent induction of RPE cell loss across different experimental groups, which is essential for reliable and interpretable research results. Unlike other methods of RPE removal, such as surgical removal, chemical ablation^12^, or high-power, longer pulse duration laser photocoagulation methods^35–39^, nanosecond laser-induced RPE removal is a non-invasive and localized approach. It does not require the mechanical removal of tissue, long exposure time, or the use of potentially toxic chemicals. Therefore, nanosecond laser reduces the risk of side effects and allows for targeted manipulation of RPE cells. In a previous study, we demonstrated the application of laser photocoagulation to induce RPE injury for the study of progenitor cell tracking^17^. However, a limitation of this previous study is that this millisecond laser photocoagulation method not only removes RPE tissue but also significantly impacts the entire retinal architecture and affects retinal function.

This model can be used to investigate the effects of RPE loss on retinal structure, function, and degeneration in different disease models, allowing for a better understanding of disease mechanisms and potential therapeutic interventions.

The nanosecond laser-induced RPE removal is a relatively rapid procedure compared to other methods of inducing RPE loss, such as genetic manipulation or chemical treatments, which may require longer time frames for effects to manifest. This allows for shorter experimental timelines and potentially more efficient research studies. This could serve as an animal model of retinal degeneration diseases such as geographic atrophy in age-related macular degeneration (*AMD*), where RPE loss is a key pathological feature, without inducing inflammation and PVR that may confound results. Therefore, findings from studies using this model may help accelerate potential therapeutic strategies.

The nanosecond laser-induced large RPE damage model provides a valuable tool for investigating the role of RPE cells in retinal degeneration and may contribute to the development of new therapeutic approaches for different retinal degeneration diseases. While this method offers several advantages for the study of retinal degeneration diseases, there are also limitations to consider, including its limited relevance to complex retinal diseases, acute and localized nature of RPE loss, normal surrounding RPE cells and background cellular environment, and limited translational relevance. Careful consideration of these limitations is important for the appropriate interpretation and application of research findings from this model. A further challenge associated with this method is the potential for long-term effects on retinal tissues due to the high laser energy used in creating the RPE damage model. A subsequent longitudinal assessment will be conducted to investigate any systemic toxicity to the retina. Minor changes are noted in the photoreceptor layer and adjacent outer retinal layers on detailed histological analysis, which could represent the role of the RPE in photoreceptor integrity, but these changes are much less than changes with longer pulse duration laser ablation and other animal models.

Although imaging guidance has been applied to improve the treatment of nanosecond laser and tracking of stem cells, it is difficult to distinguish the transplanted stem cells from native RPE cells. No significant change in retinal thickness was detectable using OCT imaging, indicating that the laser and stem cells did not damage or alter the neurosensory retina but rather were selective for the RPE. Further research and longer-term follow-up studies could better elucidate the long-term implications.

To date, the majority of studies focusing on selective retina therapy (SRT) or 2RT for AMD have concentrated on short-term outcomes and safety^32^. Burri et al*.* have provided valuable insights into the immediate effects of laser treatment on RPE degeneration progression. There is an important need to delve into the long-term repercussions. Late-stage AMD is characterized by extensive loss of RPE cells and damage to photoreceptors. This necessitates more comprehensive research to thoroughly understand the enduring impacts of laser therapy. In our study, we aimed to investigate the potential side effects of nanosecond laser treatment on neighboring neurosensory tissues, such as photoreceptors, by monitoring changes in retinal structure for up to 14 days. Encouragingly, we found no evidence of alterations in the retinal architecture during this timeframe. A future study could consider evaluating long-term changes over three months to see regenerative or compensatory changes in the retinal tissue following removal of RPE cells. Such an extended study duration would shed light on how the retina adapts and responds to the absence of RPE cells. The insights gained from this study could identify regenerative mechanisms and assess their sustainability over time. Ultimately, this knowledge holds the potential to drive the development of more effective interventions for late-stage AMD patients, thereby enhancing their quality of life and preserving their precious vision.

In conclusion, this study provides evidence that multimodal photoacoustic microscopy (PAM) and OCT can be a promising technique for guiding a reproducible nanosecond laser-induced RPE damage model in pigmented rabbits. The combined use of PAM and OCT allows for real-time imaging and monitoring of RPE damage, which may aid in the development of laser-induced RPE damage models for further research and potential clinical applications. Further studies and optimizations are warranted to fully establish the feasibility and reproducibility of this technique in animal models. The findings of this study suggest that the use of multimodal PAM and OCT for guiding reproducible nanosecond laser-induced RPE damage in pigmented rabbits could be a valuable approach in future studies related to retinal degenerative diseases and their treatment.

## Materials and methods

### Animal preparation

Dutch Belted rabbits were selected for this experiment due to the strong light absorption of melanosomes or melanin pigment in the RPE layer. In addition, the rabbit eye is similar to that of the human eye in axial length and other properties and can allow for subretinal injection to be performed. In these experiments, we used 12 rabbits (3–5 months old, 1.9–2.5 kg). The rabbits were obtained from Covance (Covance, Madison, Wisconsin, USA). All of the experimental procedures were performed under the guidance of the ARVO Statement for the Use of Laboratory Animals in Ophthalmic and Vision Research and approved by the Institutional Animal Care & Use Committee (IACUC) of the University of Michigan (Protocol PRO00010388). The study is reported in accordance with ARRIVE guidelines. All of the animals utilized in this study were treated with laser to recreate the laser-treated RPE layer and received subretinal injection of suspension hiPSC-RPE cells labeled with ICG dye. The rabbits were kept in a 12 h light–dark cycle room, and the room temperature was maintained at 21 ℃. All animals were allowed access to water and administered standard laboratory food freely. The animals were anesthetized with ketamine (40 mg/kg) and xylazine (5 mg/kg). The rabbit eyes were dilated by tropicamide 1% ophthalmic and phenylephrine hydrochloride 2.5% ophthalmic. Before laser or imaging, 0.5% topical tetracaine or proparacaine was given for topical anesthesia. To avoid corneal dehydration, a drop of phosphate-buffered saline (Gibco, Life Technologies; Grand Island, NY, USA) was provided every minute during the experiment. For imaging and laser treatment, the anesthetized rabbits were placed on two stabilization platforms to minimize motion artifacts.

### Laser-induced RPE damage procedure

In order to induce a laser-damaged RPE monolayer, we used a tunable OPO laser system pumped by Nd:YAG laser (NT-242, Ekspla, Vilnius, Lithuania). The laser incident light wavelength can be adjusted from 405 to 2600 nm with a pulse duration of 3–6 ns. We selected the excitation wavelength of 532 nm as the light source for the treatment because melanosomes in RPE cells readily absorb photons at this wavelength more than longer wavelengths as reported by Jacques et al*.*^40^. The maximum laser energy is about 250 µJ, and we selected the different laser energies of 200, 400, and 800 nJ. The emission light from the laser was filtered, collimated, and co-aligned with the light path from the OCT system to allow for imaging-guided treatment in real-time as shown in Fig. 1. The laser spot has an estimated diameter of 20 μm on the retina. We used a CCD camera integrated with the OCT system to visualize the fundus of the eye. The animals were treated with varying laser energies. Square multi-spot patterns were implemented with the area ranging from 2 × 2 mm^2^ to 5 × 5 mm^2^.

### Imaging evaluation of RPE removal area

A comprehensive ophthalmic examination was performed on all the treated eyes to evaluate any unexpected side effects to the anterior segment of the eye, including the cornea, lens, anterior chamber, conjunctiva, eyelid, and limbus. This procedure was conducted using a slip-lamp system (Zeiss SL130 Slit lamp, Carl Zeiss Meditec AG, Jena, Germany).

Color fundus photography, red-free, FAF, FA, ICGA, OCT, and PAM imaging were employed to evaluate the treated RPE removal lesions. The Topcon 50EX system (Topcon Corporation, Tokyo, Japan) with an integrated Canon (Tokyo, Japan) EOS 5D camera (5472 × 3648 pixels and a pixel size of 6.55 µm^2^) was used to obtain color photography with a field of view of 50^°^. Five different locations of the eye were captured including the temporal medullary ray, the nasal medullary ray, the superior retina above the optic disc, the inferior retina below the optic disc, and the optic nerve. Then, I2K Retina software was applied to process a montage image.

The Topcon system was used to obtain red-free images, FAF, FA, and ICGA by changing the internal excitation and emission filters for each. The excitation filter for red-free is 510 nm, FAF is 540 nm, FA is 485 nm, and ICGA is 769 nm. For FA and ICGA imaging, a dose of 0.2 mL (100 mg/mL) of fluorescein sodium (Akorn, Lake Forest, IL, USA) or 0.2 mL (2.5 mg/mL) of ICG dye (HUB Pharmaceuticals LLC, Patheon, Italy) was intravenously injected in each animal through the marginal ear vein. Serial images were obtained after fluorophore injection. The FA images were captured both prior to and immediately after laser irradiation, and then at 2 h post treatment following ICGA administration. Subsequently, a longitudinal study was conducted for a period of up to 14 days. The serum half-life in humans of indocyanine green is 3–4 min and fluorescein sodium is 23.5 minutes^41,42^. Thus, after 2 h, an estimated 5.8*10^−9^% of ICG and 2.9% of fluorescein would remain. With fluorescein and indocyanine green dye small molecules, most is clear from the body via urine by 2 h post injection. Thurs, we started another injection after 2 h following the first dose.

Cross-sectional OCT images were obtained using the Ganymede-II-HR system (Thorlabs, Newton, NJ, USA). Two super luminescent diodes (λ1 = 846 nm and λ2 = 932 nm) were used to acquire 2D OCT images with a scanning rate of 36 kHz, scan angle ± 10.6°, and beam diameter 5.5 mm. The system provides high resolution and highly detailed retinal structure with an aerial axial resolution of 4.0 µm and lateral resolution of 3.8 µm. To achieve the large-treatment area of 5 × 5 mm^2^, we used a galvanometer scanning along the x- and y-axes. Different treatment patterns could be achieved using the developed scanning software. During the laser treatment, 3D volumetric OCT data was recorded with a resolution of 512 × 512 pixels and scanning speed of 5 kHz and rendered to evaluate the treatment effects.

PAM images were achieved using multimodal PAM and OCT imaging systems^18,36–39,43–45^. An optical parametric oscillator (OPO) laser system was used to generate PA signals (NT-242, Ekspla, Vilnius, Lithuania). Two different excitation wavelengths of 578 and 700 nm were utilized to visualize the retinal microvasculature and to distinguish the distribution of stem cells after transplantation. The PA signal was detected by a custom-built needle-shaped 27 MHz ultrasonic transducer (Optosonic Inc, Arcadia, CA, USA), and the 3D volumetric visualization images were rendered using Amira software. The spot size of the laser in the retina is measured to be 20 µm as reported by previous studies^17,18,35–39,46^.

### Stem cell culture

hiPSC cells were obtained from LAgen Laboratories LLC (Line 006-BIOTR-0001 Clone 1). The cells were cultured on 6-well plates in mTESR1 media. The cells’ media was replaced every week and passaged when the cell confluence reached 50–75%. hiPSC were differentiated into RPE (hiPSC-RPE) as described in previous publication^47^. Then, the hiPSC-RPE cells were cultured for up to 6 months with the formation of pigmentation and cobblestone morphology. Afterward, the cells were treated with ICG conjugated with arginine-glycine-aspartate (RGD) peptides at a final concentration of 100 µg/mL. At 24 h post-treatment, the cells were trypsinized using Trypsin-EDTA (0.25%), centrifuged, and suspended in fresh media for the injection.

### Stem cell transplantation

At day 14 post-laser treatment, the treated eyes received subretinal injection of hiPSC-RPE cells labeled with ICG dye. Rabbits were first anesthetized with ketamine and xylazine and the pupils were dilated. A tunnel was created 3.5–4 mm from the limbus to avoid cataract formation. A contact lens (Volk H-R Wide Field, laser spot 2 × magnification, Volk Optical Inc., Mentor, OH, USA) coated with Gonak gel (Akorn, Lake Forest, IL, USA) was mounted directly on the cornea and the targeted location (RPE damage areas) were monitored under a microscope. 50 µL of hiPSC-RPE suspension cells (2 × 10^6^ cells/mL) were prepared using a 50 µL syringe (Hamilton, Germany). A 30-gauge needle was gently inserted into the scleral tunnel and reached the retina. When the color at the surface of the retina was observed to change to white, the cells were gradually delivered into the subretinal space via a 30-gauge needle (Hamilton, Germany). Following the subretinal administration, fluorescence and OCT images were acquired immediately and longitudinally for up to 14 days of follow-up. PAM imaging was performed before and at day 14 post-cell transplantation.

### Histological and immunohistochemical analysis

All the animals were euthanized at day 14 post-stem cell transplantation to assess the RPE damage and to evaluate the potential of the RPE removal model for stem cell therapy. The rabbits were euthanized by intravenous injection of euthanasia solution at a dose of 0.22 mg/kg (Beuthanasia-D, VetOne, MWI Animal Health, Boise, ID, USA). The eyeballs were harvested and pre-fixed in Davidson’s fixative solution for 24 h. The fixed eyes were sequentially transferred to 50% for 8 h and 70% for another 24 h. Then the samples were dissected into halves and embedded in paraffin. The embedded samples were sectioned into 4–6 µm thick sections and stained with hematoxylin and eosin (H&E).

To visualize the grafted hiPSC-RPE cells, IHC was performed using anti-RPE-65 against rabbit RPE65 (MAB5428, MilliporeSigma, MA, USA) and anti-NuMA antibodies against human NuMA (ab84680, Abcam, MA, USA). To visualize the outer segments of photoreceptors, immunofluorescence staining was conducted with the anti-Rhodopsin antibody (ab190307, Abcam, MA, USA). The H&E and immunofluorescence images were captured by a Leica DM6000 light microscope (Leica Biosystems, Nussloch, Germany).

### Statistical analysis

In this study, all experiments were performed at least three times for each condition. To evaluate potential differences between the NZW and DB groups, an analysis of variance (ANOVA) was conducted using Origin software. The findings were reported as mean ± standard deviation (SD), with p-values below 0.05 considered statistically significant.

### Supplementary Information

Supplementary Figure S1.

## Data Availability

The datasets used during the current study available from the corresponding author on reasonable request.
