# Supplementary material for: Selective nanosecond laser removal of retinal pigment epithelium for cell therapy
Source: Sci Rep. 2024 Aug 21;14:19457. doi: 10.1038/s41598-024-69917-z (PMC11339075; doi:10.1038/s41598-024-69917-z)
Supplement: Supplementary file 1 — Supplementary Figure S1. [file 41598_2024_69917_MOESM1_ESM.doc]

**Supplementary Information for Figures**

**Selective Nanosecond Laser Removal of Retinal Pigment Epithelium for Cell Therapy**

**­­­ Van Phuc Nguyen1, Athanasios Karoukis1, Justin Hu1, Dongshan Yang3, Abigail Fahim1, Xueding Wang1, and Yannis M. Paulus1,2***

1Department of Ophthalmology and Visual Sciences, University of Michigan, Ann Arbor, MI 48105, USA

2Department of Biomedical Engineering, University of Michigan, Ann Arbor, MI 48105, USA

3Center for Advanced Models for Translational Sciences and Therapeutics, University of Michigan, Ann Arbor, MI 48109, USA

*Corresponding Authors:

Yannis M. Paulus, M.D., F.A.C.S.

Department of Ophthalmology and Visual Sciences

Department of Biomedical Engineering

University of Michigan

1000 Wall Street

Ann Arbor, MI 48105, USA

Email Address: [ypaulus@med.umich.edu](mailto:ypaulus@med.umich.edu)

**Supplementary Figures**


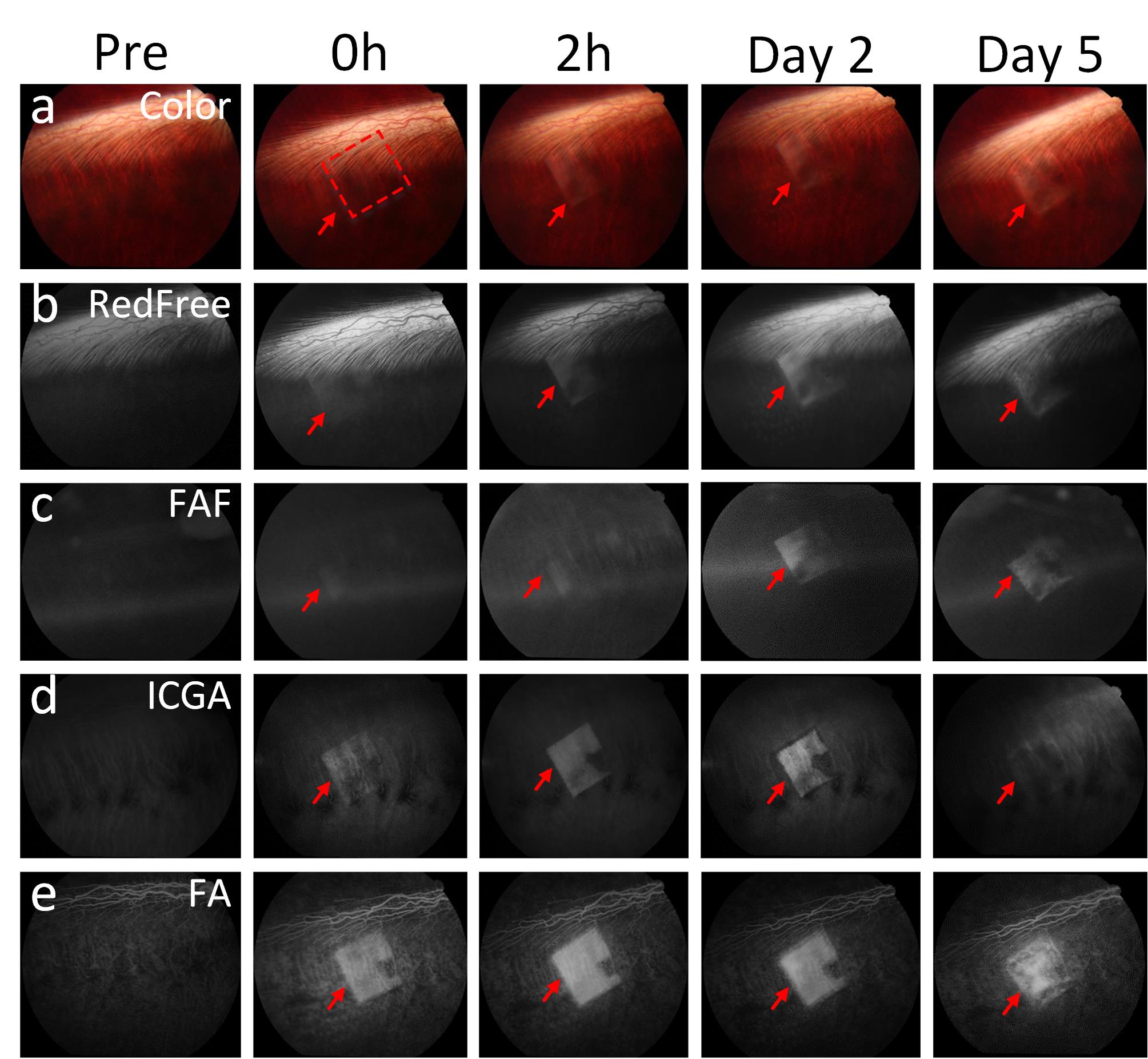


**Supplementary Figure S1.** **Visualizing RPE degeneration *in vivo* at 800 nJ energy levels.** (a) Sequentially captured color fundus photographs obtained at different time points: pre- and post- laser irradiation at 800 nJ. Highlighted by red dashed squares and arrows, depicting the laser treatment zone. (b) Red-free fundus images. (c) Fundus autofluorescence (FAF) images illustrating the progression of RPE cell degeneration following laser treatment over time. (d) Indocyanine green angiography (ICGA) images. Prominently delineated hyperfluorescent regions (window defects) as a clear indication of RPE cell loss. (e) Fluorescein angiography (FA) images. These images were obtained at late phase to visualize RPE cell demise.
